# *Brachypodium distachyon* BdPP2CA6 Interacts with BdPYLs and BdSnRK2 and Positively Regulates Salt Tolerance in Transgenic *Arabidopsis*

**DOI:** 10.3389/fpls.2017.00264

**Published:** 2017-02-28

**Authors:** Fan Zhang, Qiuhui Wei, Jiaochun Shi, Xia Jin, Yuan He, Yang Zhang, Qingchen Luo, Yuesheng Wang, Junli Chang, Guangxiao Yang, Guangyuan He

**Affiliations:** The Genetic Engineering International Cooperation Base of Chinese Ministry of Science and Technology, Key Laboratory of Molecular Biophysics of the Ministry of Education, College of Life Science and Technology, Huazhong University of Science and TechnologyWuhan, China

**Keywords:** *Brachypodium distachyon*, abiotic stress, salinity, ABA, PP2C

## Abstract

The phytohormone abscisic acid (ABA) is essential in plant responding to biotic and abiotic stresses. Although ABA signaling model is well established in *Arabidopsis*, ABA receptor PYL family and clade A PP2C subfamily are not yet characterized in monocot model plant *Brachypodium distachyon*. In this study, we identified 12 *PYLs* and 8 clade A *PP2Cs* from *B. distachyon* genome and successfully cloned 12 *PYLs* and 7 clade A *PP2Cs*. Bioinformatic and expression analyses showed that most of the identified genes respond to several signal molecules and abiotic stresses. Protein–protein interaction analysis revealed that many BdPYLs and BdPP2CAs participate in the classic ABA-PYL-PP2C-SnRK2 signaling pathway. A clade A PP2C, designated BdPP2CA6, interacted with BdPYL11 in the absence of ABA and localized in nucleus. Most clade A PP2C members from *Arabidopsis* showed negatively regulation in ABA signaling pathway, whereas *BdPP2CA6*-overexpression transgenic *Arabidopsis* showed ABA hypersensitive phenotype, resulting in enhanced stomatal closure and salinity tolerance. Our results indicate that *BdPP2CA6* positively regulates ABA and stress signal pathway in transgenic *Arabidopsis* plant seedlings.

## Introduction

Plants are constantly challenged by diverse biotic and abiotic stresses throughout their lifetime. Complex stress-related signal transduction pathways have been evolved to cope with those biotic and abiotic stresses (Bohnert et al., 2006). Abscisic acid (ABA) is essential in plants responding to biotic and abiotic stresses and ABA signaling model is well established in model plant species *Arabidopsis* (Lee and Luan, 2012). Type 2C phosphatase (PP2C) is a kind of protein phosphatase that dephosphorylates Ser/Thr residues (Cohen, 1989). Clade A PP2C is a subgroup of PP2C family that inhibits ABA signaling in *Arabidopsis* (Lee and Luan, 2012). Core ABA signaling module is composed of three main components: PYR/PYL/RCAR (pyrabactin resistance/PYR-like protein/regulatory component of ABA receptor), clade A PP2C, and SnRK2 (SNF1-related protein kinase 2) (Fujii et al., 2009; Ma et al., 2009; Park et al., 2009). In this model, PP2Cs physically interact with SnRK2s and inhibit SnRK2 kinase activities in the absence of ABA. In the presence of ABA, an ABA molecule binds to ABA receptor PYR/PYL/RCAR to form a ternary complex PYR/PYL/RCAR-ABA-PP2C, thus causing the release of SnRK2s from PP2Cs. Subsequently, SnRK2s phosphorylate and activate downstream targets to respond to external stresses (Klingler et al., 2010; Lee and Luan, 2012; Osakabe et al., 2014).

To date, plenty of studies have revealed involvement of PP2Cs and PYR/PYL/RCARs in stress signaling pathways in plants. By inhibiting PP2C activities, PYR/PYL/RCARs function positively in major abiotic stress signal pathways. Overexpressing of PYL4^A194T^, a mutant that can form stable complex with PP2CA without exogenous ABA, reduces stomatal conductance and enhances water use efficiency in transgenic seedlings in comparison with wild type (WT) or *PYL4*-overexpressing seedlings, thus enhancing drought and dehydration tolerance (Pizzio et al., 2013). PYL5 is a positive regulator of ABA signaling and enhances drought resistance through inhibition of HAB1 (Santiago et al., 2009). PYL9 inhibits ABI1, HAI1, HAI2, HAI3, HAB1, HAB2, and PP2CA, and promotes leaf senescence and resistance to extreme drought stress in *Arabidopsis* (Zhao et al., 2016). Studies also demonstrate that clade A PP2Cs in *Arabidopsis*, such as ABI1, ABI2, PP2CA, and HAB1, negatively regulate ABA signaling, while their loss-of-function mutants display improved tolerance to abiotic stress such as salinity and drought (Merlot et al., 2001; Saez et al., 2004, 2006; Zhang et al., 2013). However, recently a clade A PP2C gene isolated from rice, namely *OsPP108*, is found to positively regulate abiotic stress signaling through ABA independent pathway, indicating that clade A PP2Cs have multiple functions in stress signal pathways (Singh et al., 2015).

By now, putative ABA signaling genes in monocot such as rice, barley, and maize have been identified. The rice PYR/PYL/RCAR family has 12 members and its PP2C superfamily consists of 78 members, 10 of which belong to clade A subfamily (Xue et al., 2008; He et al., 2014). In barley, nine *HvPYR/PYLs* and six clade A *HvPP2Cs* have been identified (Seiler et al., 2014). While in maize, 11 *ZmPYLs* and 5 clade A *ZmPP2Cs* have been identified (Fan et al., 2016). However, PYL family and clade A PP2C subfamily have not yet been characterized in monocot grass species *Brachypodium distachyon*, which is an excellent model species for functional genomics research in temperate grasses. In this study, we identified 12 PYL and 8 clade A PP2C genes from *B. distachyon*, and analyzed their chromosomal distributions and expression patterns. Additionally, interaction analyses were performed between PP2Cs and PYR/PYL/RCARs or PP2Cs and SnRK2s to determine their interaction network in response to abiotic stresses. Furthermore, functional analyses of BdPP2CA6 revealed its positive regulatory role in response to salinity through ABA dependent pathway. These analyses are helpful for understanding core ABA signaling in *B. distachyon* and may shed light on functional characterization of PP2Cs in monocot crop plants, such as wheat.

## Materials and Methods

### Plant Materials

The *B. distachyon* Bd21 was cultured and used for gene cloning and expression analysis. The *Arabidopsis thaliana* ecotype Colombia (Col-0) was used as WT control. The pSN1301-BdPP2CA6 plasmid was transformed into *Arabidopsis* using *Agrobacterium tumefaciens* strain EHA105 as described previously (Clough and Bent, 1998). *Arabidopsis* transformed with pSN1301 empty vector was used as vacant vector control (VC). Seeds of transformed *Arabidopsis* were selected using MS medium supplemented with 20 mg/L hygromycin. Homozygous lines of T_3_ and T_4_ generations were used for further analysis.

### Plant Growth Conditions and Treatments

All plant materials were grown under a 14 h light/10 h dark cycle and 70% relative humidity at 22°C. For organ-specific expression assay, different organs including root, stem, leaf, and caryopsis were taken, respectively, from 3-week-old *B. distachyon* plants. For abiotic stress treatments, 3-week-old *B. distachyon* were treated with 100 μM ABA, 200 mM NaCl, or 20% (w/v) PEG-6000, respectively, and then leaves were sampled at 0, 1, 3, 6, 12, and 24 h after treatments.

### Identification and Analysis of *BdPYLs* and Clade A *BdPP2Cs*

To identify *PYLs* and clade A *PP2Cs* in *B. distachyon*, PYL and clade A PP2C gene sequences of *Arabidopsis* were downloaded from TAIR database^1^, which were then used to do BLAST searches against Ensembl Plants database^2^. All resulting proteins were confirmed to contain certain domains using Pfam database^3^. Identified sequences of *B. distachyon* were aligned using ClustalX2.0 with default parameters (Larkin et al., 2007). Phylogenetic trees were constructed using bootstrap neighbor-joining (NJ) method and bootstrap analysis (1,000 replicates) by MEGA6.0 (Tamura et al., 2013). Promoter sequences and exon-intron data were obtained from Plaza database^4^. Abiotic stress-responsive *cis*-elements in promoters were analyzed using PlantCare database^5^. Expression heatmaps of *BdPP2CAs* and *BdPYLs* were constructed as described previously (Chen L. et al., 2016).

### Plasmid Construction

A total of 7 *BdPP2CAs* and 12 *BdPYLs* were amplified by RT-PCR. For yeast two-hybrid assay, ORFs of *BdPP2CAs* and *BdPYLs* were amplified from *B. distachyon* cDNA and cloned into vectors pGBKT7 and pGADT7, respectively. The pGADT7-BdSnRK2.2, pGADT7-BdSnRK2.3, and pGADT7-BdSnRK2.6 vectors were obtained from our laboratory (Wang et al., 2015). The ORFs of the *AtSnRK2.2, AtSnRK2.3*, and *AtSnRK2.6* were amplified from *Arabidopsis* Col-0 WT cDNA and were cloned into vector pGADT7. For BiFC assay, ORFs of *BdPP2CA6* were cloned into vector pUC-SPYNE, while *BdPYL11*, and *AtSnRK2.3* were cloned into vector pUC-SPYCE. For GFP fusion protein expression, 35S::BdPYL11::GFP, 35S::BdPP2CA6::GFP, and 35S::AtSnRK2.3::GFP were constructed. For plant transformation, ORF of *BdPP2CA6* was cloned into vector pSN1301 driven by *CaMV 35S* promoter.

All primers used are listed in Supplementary Table S2 and all amplified fragments were confirmed by sequencing. All accession numbers of the genes mentioned in manuscript are listed in Supplementary Table S3.

### Yeast Two-Hybrid Assay

Each pair of constructed plasmids were co-transformed into yeast strain AH109 following the manufacturer’s protocol (Clontech Inc., USA) and subsequently plated onto SD minimal medium without leucine and tryptophan (SD/-Leu-Trp). Then yeast cells were transferred to specific selection media for yeast growth assessments.

### Subcellular Localization and BiFC Assay

The constructs of BdPYL11-GFP and BdPP2CA6-GFP were transformed, and constructs of BdPP2CA6-YNE and BdPYL11-YCE or AtSnRK2.3-YCE were co-transformed into onion epidermal cells by using biolistic method (Biolistic PDS-1000/He Particle Delivery System, Bio-Rad, USA), respectively (Hanson and Kohler, 2001). Fluorescence was observed by fluorescence microscope (IX71, Olympus, Japan) 1 day after transformation. DAPI (4′,6-diamidino-2-phenylindole) staining was performed as described previously (Sun et al., 2015).

### RNA Extraction and qRT-PCR Analysis

Total RNAs were isolated using Plant RNA Extraction Kit (ZOMANBIO, China). The cDNAs were obtained by using FastQuant RT Kit (TIANGEN, China). The qRT-PCR analysis was performed using SYBR Green Kit (TIANGEN, China) on a real-time PCR machine (CFX96^TM^ Real-time Detection System, Bio-Rad, USA). Gene-specific primer sets were listed in Supplementary Table S2 and some of them were designed according to previous studies (Fujii et al., 2007; Feng et al., 2014; Chen Z.H. et al., 2016). All amplified fragments were confirmed by sequencing. The qRT-PCR analysis was performed as described previously (Hu et al., 2013).

### Stomatal Aperture Analysis

For stomatal aperture measurement, leaves of 4-week-old WT and transgenic plants were collected and incubated in solution containing 30 mM KCl, 10 mM MES-Tris, and 50 μM CaCl_2_ (pH 6.15) with light for 2 h. Then 50 μM ABA or 150 mM NaCl was added to the solution. After 2 h treatments, leaf samples were examined using microscope (IX71, Olympus, Japan). Widths and lengths of stomata were measured to figure out stomatal apertures.

### Stress Tolerance Assay

For ABA treatment, 7-day-old seedlings germinated on 1/2 MS medium were transferred to MS medium or MS media supplemented with 1, 5, or 10 μM ABA for 10 days. For salinity stress tolerance assay at early development stage, 7-day-old seedlings were transferred onto MS medium or MS medium supplemented with 150 mM NaCl for 10 days. For physiological index measurement and qRT-PCR analysis, 4-week-old seedlings cultured in soil were watered with or without 150 mM NaCl for 10 days and then leaf samples were collected. Survival rates of the treated *Arabidopsis* seedlings were measured 35 days post NaCl treatment and pictures of the seedlings were taken 30 days post NaCl treatment.

### Ion Content Measurement

For ion content determination, treatment of plants and sampling of leaves were performed as described previously with slight modification (Deng et al., 2013). One-week-old seedlings culturing on MS medium were transferred onto MS medium supplemented with 150 mM NaCl for 1 week and then leaves were sampled. Measurement was carried out by using Atomic Absorption Spectrometry (ContrAA 700, Analytik Jena, Germany).

### Physiological Index Measurement

Measurements of ion leakage (IL), H_2_O_2_, proline, chlorophyll, and malondialdehyde (MDA) content were performed as described previously (Huang et al., 2015).

### Statistical Analysis

Statistical analyses were performed using Excel (Microsoft, USA) and SPSS (IBM, USA). Figures were plotted by using OriginPro (OriginLab, USA).

## Results

### Identification of BdPYL Family and Clade A BdPP2C Subfamily in *B. distachyon*

**FIGURE 1:**
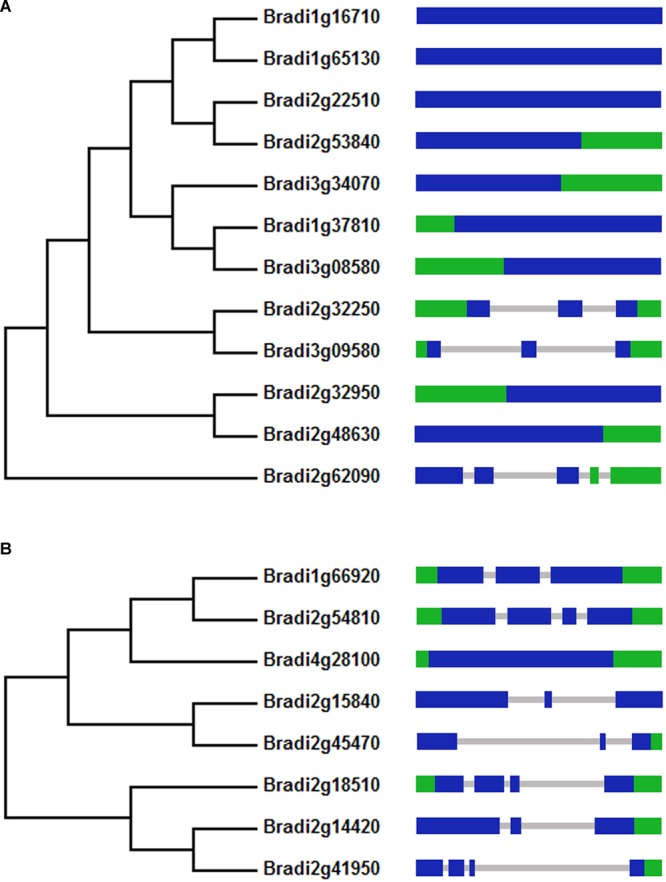
**Phylogenetic and exon-intron analyses of *BdPYLs* (A)** and *BdPP2CAs* **(B)**. The green area represents UTR exon, the blue area represents exon, and the gray area represents intron.

To identify BdPYL and clade A BdPP2C proteins from *B. distachyon*, BLAST searches were performed. Twelve putative BdPYL proteins and eight putative clade A BdPP2C proteins were identified from the *B. distachyon* genome. Phylogenetic analyses were performed to construct phylogenetic trees by using full length amino acid sequences of BdPYL family or clade A BdPP2C subfamily, and the results showed that key domains of BdPYL proteins or clade A BdPP2C proteins are evolutionary conserved (**Figure 1**).

### Chromosomal Locations of *BdPYLs* and Clade A *BdPP2Cs*

To analyze the genomic distribution of *BdPYLs* and clade A *BdPP2Cs*, their approximate positions on each chromosome were marked. The results showed that six *BdPYLs* distributed on chromosome 2 and the rest members were localized on chromosomes 1 and 3, while six clade A *BdPP2C*s distributed on chromosome 2 and the rest members were mapped on chromosomes 1 and 4 (Supplementary Figure S1). All *BdPYLs* and clade A *BdPP2Cs* were designated according to their distribution on chromosomes as listed in Supplementary Table S1.

### Analyses of Gene Structures and *Cis*-elements in Promoters

The exon–intron structures of *BdPYLs* and *BdPP2CAs* were also analyzed (**Figure 1**). For *BdPYL* family, only three *BdPYLs* contains two or more introns and the rests contain no intron at all. For *BdPP2CA* subfamily, all members except *BdPP2CA8* have two or three introns. These conserved intron and extron numbers in both *BdPYL* family and *BdPP2CA* subfamily support their close evolutionary relationships.

To identify abiotic stress-responsive *cis*-elements in promoters, 1,500 bp sequences upstream of *BdPYL* and *BdPP2CA* CDSs were obtained, and several abiotic stress-responsive *cis*-elements (ABRE, CE3, CGTCA-motif, GARE-motif, HSE, TGACG-motif, MBS, TCA-element) were identified. The results demonstrate that 50% of the *BdPYLs* contain ABRE elements in their promoter regions and only *BdPP2CA5* has no ABRE element in its promoter region, implying that many *BdPYLs* and *BdPP2CAs* maybe ABA responsive. Results also show that these genes have one or more additional stress-responsive *cis*-elements in their promoter regions, suggesting their potential involvements in various stress responses (Supplementary Table S1).

### Expressions of *BdPP2CA6* and *BdPP2CA8* Are Responsive to Salt or Drought Treatments

To analyze the potential functions of *BdPYLs* and *BdPP2CAs* under environmental stresses, public microarray data were obtained to analyze their expressions under cold, heat, drought, and high-salinity conditions (Priest et al., 2014). The results showed that expressions of most *BdPYLs* were not obviously changed under stress treatments, while *BdPP2CA6* was significantly up-regulated under high-salinity stress and *BdPP2CA8* was significantly up-regulated under drought stress (Supplementary Figure S2).

### *BdPYLs* and *BdPP2CAs* Cloning and Yeast Two-Hybrid Assay

**FIGURE 2:**
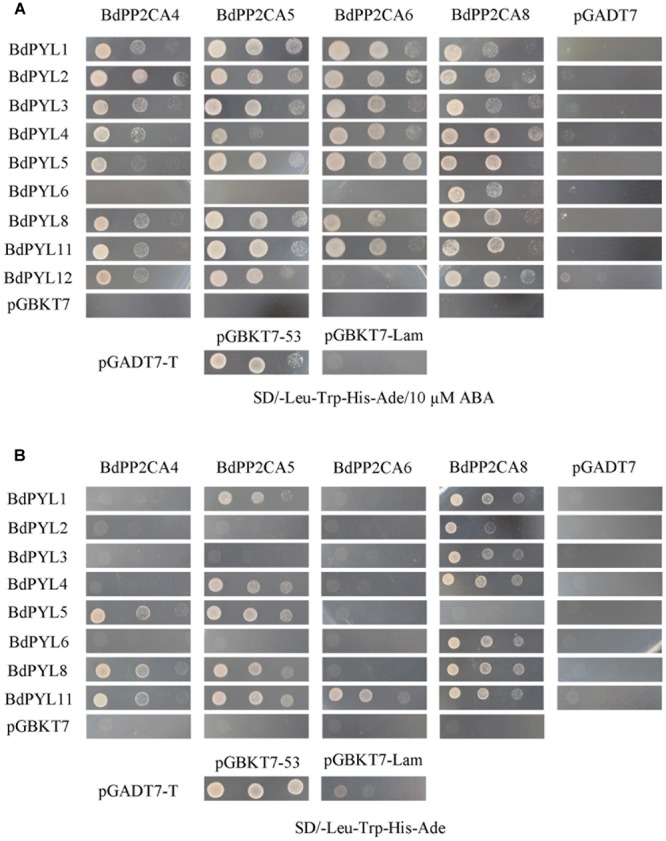
**Interaction analyses of BdPYLs-BdPP2CAs.** The yeast strains AH109 transformed with different combinations of plasmids were cultivated on selective medium **(A)** SD/-Trp-Leu-His-Ade/10 μM ABA or **(B)** SD/-Trp-Leu-His-Ade. Interactions between SV40-T and p53 or SV40-T and human lamin C were set as positive or negative control, respectively. Yeast strains were assessed by different dilution rates (1, 1/10, and 1/100). Data of those BdPP2CAs/BdPYLs which could not interact with any BdPYLs/BdPP2CAs were not shown.

Although we tried hard to clone *BdPP2CA3* from the *B. distachyon* cDNA, we failed to clone the full length of it. The rest 7 *BdPP2CAs* and all identified *BdPYLs* were successfully cloned. In *Arabidopsis*, ABA-bound PYLs interact with clade A PP2Cs and inhibit their activities (Melcher et al., 2009; Miyazono et al., 2009; Yin et al., 2009). To investigate their interaction networks in *B. distachyon*, we examined the interactions between BdPP2CAs and BdPYLs in yeast two-hybrid assay. Data of those BdPP2CAs/BdPYLs which could not interact with any BdPYLs/BdPP2CAs were not shown. The result showed that BdPP2CA4, BdPP2CA5, BdPP2CA6, BdPP2CA8 interact with 7 or 8 or 9 BdPYLs when supplied with exogenous ABA (**Figure 2A**). In the absence of exogenous ABA, fewer BdPYLs interact with these four BdPP2CAs (BdPP2CA4, BdPP2CA5, BdPP2CA6, and BdPP2CA8) and only BdPYL11 can interact with all of them (**Figure 2B**).

### Expression Patterns of *BdPYL11* and *BdPP2CAs*

**FIGURE 3:**
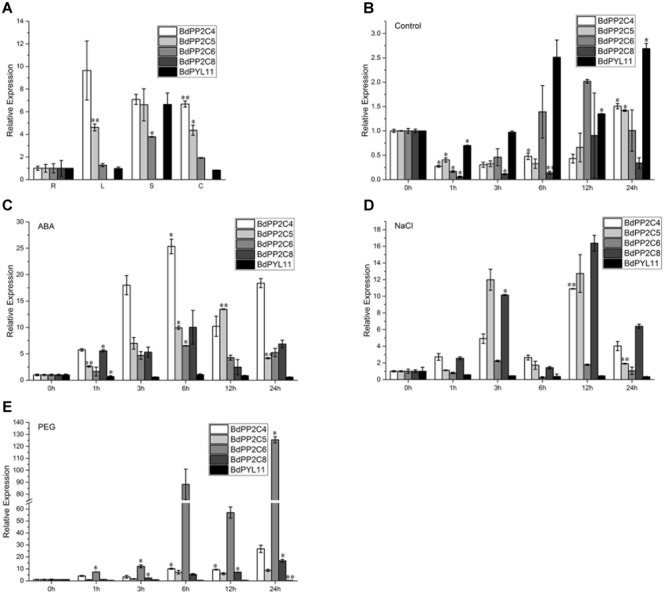
**Organ-specific expression patterns of *BdPYL11, BdPP2CA4, BdPP2CA5, BdPP2CA6*, and *BdPP2CA8* (A)**, and expression patterns of these genes under normal **(B)**, 100 μM ABA **(C)**, 200 mM NaCl **(D)**, and 20% (w/v) PEG-6000 **(E)** treatments. The organs (root, stem, leaf, and caryopsis) were represented by R, S, L, and C, respectively. Data are means ± SE of three replicates. Significances of differences are indicated as ^∗^*P* < 0.05; ^∗∗^*P* < 0.01.

As interactions between BdPYL11 and BdPP2CA4, BdPP2CA5, BdPP2CA6, or BdPP2CA8 are strong and ABA independent, qRT-PCR analyses were performed to study their potential functions in response to abiotic stresses. Organ-specific expression analysis was also carried out, and the results demonstrated that *BdPP2CA8* expressed only in roots while *BdPYL11, BdPP2CA4, BdPP2CA5*, and *BdPP2CA6* were detected in roots, as well as in leaves, stems, and caryopses (**Figure 3A**).

In *Arabidopsis*, PYLs were strongly down-regulated while clade A PP2Cs were strongly up-regulated by ABA treatment (Merlot et al., 2001; Saez et al., 2004; Santiago et al., 2009). The qRT-PCR results reveal that expression patterns of the investigated genes in *B. distachyon* are consistent with their homologs in *Arabidopsis*. *BdPYL11* was down-regulated under ABA, NaCl and PEG6000 treatments while *BdPP2CA4, BdPP2CA5, BdPP2CA6*, and *BdPP2CA8* were up-regulated (**Figures 3B–E**). These results suggest that these genes respond to various abiotic stresses.

### Interaction between BdPP2CA6 and BdPYL11 Confirmed by BiFC Assay

**FIGURE 4:**
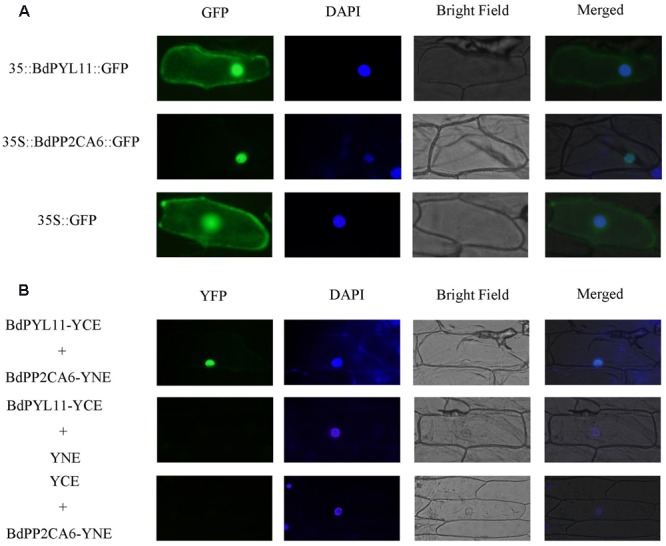
**Subcellular localization of BdPYL11-GFP and BdPP2CA6-GFP (A)**, and BiFC visualization of BdPYL11 interaction with BdPP2CA6 **(B)**. BdPYL11-GFP, BdPP2CA6-GFP, BdPYL11-YCE, and BdPP2CA6-YNE fusion proteins transiently expressed in onion epidermal cells were observed. DAPI staining for the nucleus was performed after GFP/YFP and bright field visualization. Subcellular localization of GFP was set as control. BdPYL11-YCE and YNE, BdPP2CA6-YNE and YCE were set as negative controls.

Without exogenous ABA, BdPP2CA6 only interacts with BdPYL11 in yeast two-hybrid assay. So we were more interested in interaction between these two proteins. GFP fusion vectors were constructed and transferred into onion epidermal cells to investigate their subcellular localizations. BdPYL11-GFP fusion protein was observed in the nucleus, plasma membrane, and cytoplasm, while BdPP2CA6-GFP fusion protein was observed only in nucleus (**Figure 4A**). To examine whether BdPP2CA6 interacts with BdPYL11 *in vivo*, we further carried out BiFC assay. YFP fluorescence was observed in nucleus, suggesting that BdPP2CA6 interact with BdPYL11 *in vivo* (**Figure 4B**).

### Overexpression of *BdPP2CA6* in *Arabidopsis* Affects ABA Sensitivity

*BdPP2CA6*, a clade A PP2C gene, was greatly induced by ABA, PEG6000 and NaCl treatments (**Figure 3**). BdPP2CA6 exhibited a strong interaction with BdPYL11 in the absence of ABA (**Figures 2A** and **4A**). Transient expression of BdPP2CA6-GFP fusion protein in onion epidermal cells demonstrates that BdPP2CA6 localizes only at the nucleus (**Figure 4B**). Therefore we focused on investigating BdPP2CA6 and obtained *BdPP2CA6* overexpression *Arabidopsis* plants for further analyses.

**FIGURE 5:**
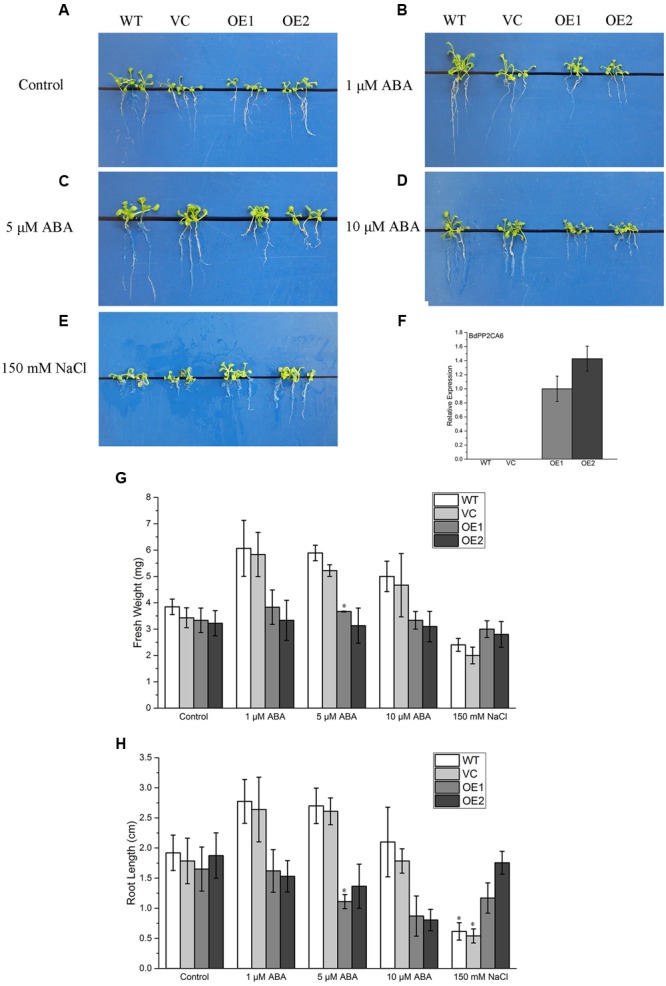
**Root elongation analyses of *BdPP2CA6*-overexpression *Arabidopsis* plants under ABA and NaCl treatment. (A–E)** Root growths of WT, VC and transgenic lines on MS medium, or MS medium supplemented with different concentrations of ABA or NaCl. **(F)** *BdPP2CA6* expression levels in WT, VC and transgenic lines. **(G)** Fresh weights of WT, VC and transgenic lines. **(H)** Root lengths of WT, VC and transgenic lines. Data are means ± SE of three replicates. Significances of differences are indicated as ^∗^*P* < 0.05.

To investigate whether overexpression of *BdPP2CA6* could affect ABA response, 7-day-old WT, VC and *BdPP2CA6*-overexpression *Arabidopsis* seedlings were placed on MS medium and MS media supplemented with different concentrations of ABA, respectively, for 10 days. ABA-mediated inhibition of root growth was affected by overexpression of *BdPP2CA6* (**Figures 5A–D**). Root elongations of *BdPP2CA6*-overexpression plants were inhibited with higher degrees than that of WT and VC by all concentrations of ABA. Fresh weights and root lengths were measured and were significantly higher for WT and VC plants than for transgenic plants at all ABA concentrations (**Figures 5G,H**). The results demonstrated that overexpression of *BdPP2CA6* enhanced ABA sensitivity at early growing stage of plants.

### BdPP2CA6 Regulates ABA-Mediated Stomatal Closure

**FIGURE 6:**
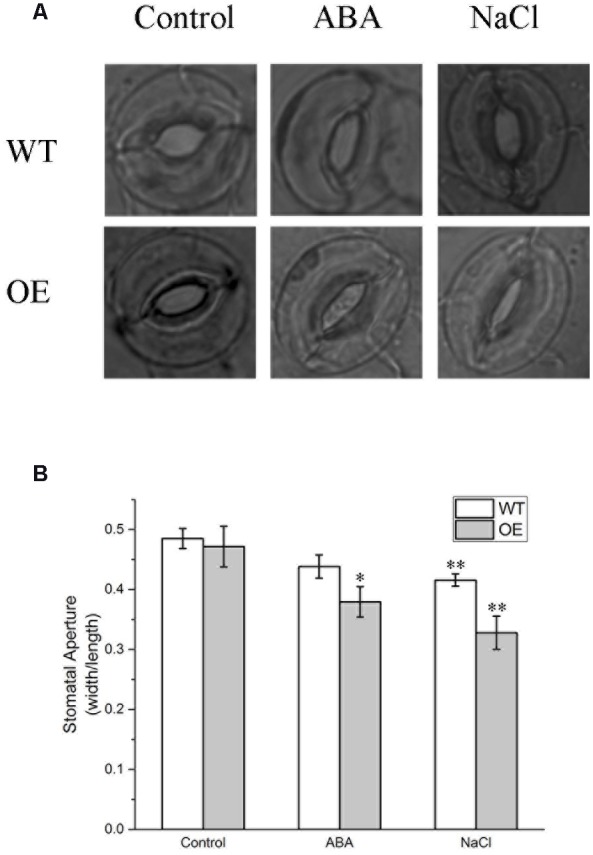
**Stomatal aperture analyses of WT and *BdPP2CA6* overexpression *Arabidopsis* plants under ABA and NaCl treatment. (A)** Representative images; **(B)** Ratio of width to length (*n* = 20–30). Data are means ± SE and significances of differences are indicated as ^∗^*P* < 0.05, ^∗∗^*P* < 0.01.

Whether BdPP2CA6 could regulate ABA-mediated stomatal closure was investigated. Under normal condition, stomatal apertures of transgenic seedlings were a little bit smaller than that of WT. After ABA treatment, stomatal apertures of all seedlings decreased, with a much more significant decrease in transgenic plants compared with WT (**Figure 6**). These results suggest that overexpression of *BdPP2CA6* promotes ABA-mediated stomatal closure.

### Overexpression of *BdPP2CA6* Increases Salinity Tolerance in *Arabidopsis* Plants

**FIGURE 7:**
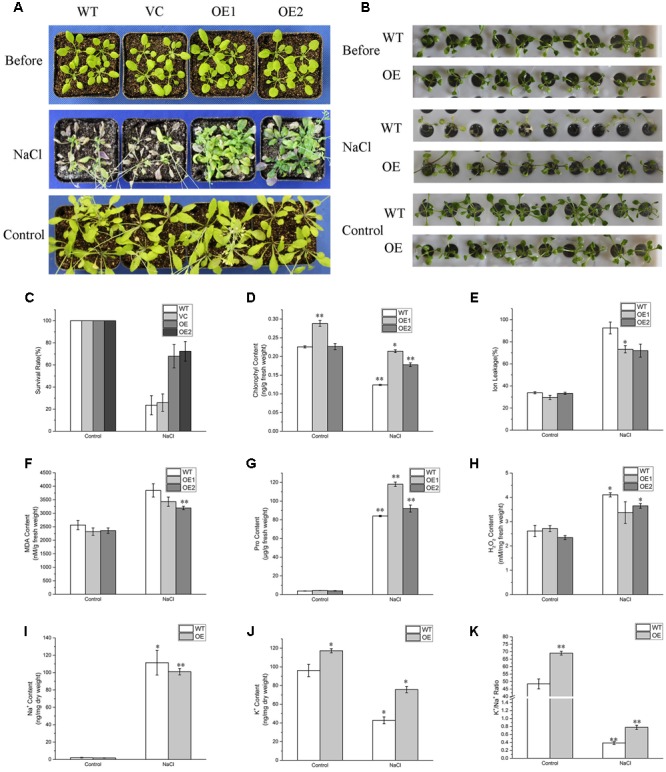
**Analyses of enhanced salinity tolerance of *BdPP2CA6*-overexpressing *Arabidopsis* seedlings. (A)** Phenotype of 3-week-old WT, VC and transgenic seedlings before NaCl treatment, 30 days post treatment, and 30 days later without treatment (control). **(B)** Phenotype of 2-week-old WT and transgenic seedlings culturing in Hoagland solution before NaCl treatment, 1 days after treatment, and 1 day later without treatment (control). **(C)** Survival rates were measured 35 days post NaCl treatment and 35 days later without treatment (control). **(D)** Chlorophyll content. **(E)** Ion leakage content. **(F)** MDA content. **(G)** Proline content. **(H)** H_2_O_2_ content. **(I)** Na^+^ content. **(J)** K^+^ content. **(K)** K^+^/Na^+^ ratio. Data are means ± SE of three replicates. Significances of differences are indicated as ^∗^*P* < 0.05, ^∗∗^*P* < 0.01.

To evaluate the effects of salinity stress on growth of *BdPP2CA6*-overexpression plants, 7-day-old WT, VC and *BdPP2CA6*-overexpression plants were placed on MS medium and MS medium supplemented with 150 mM NaCl, respectively. After 10 days, no obvious difference was discovered at post-germination developmental stage on MS media. The addition of 150 mM NaCl significantly inhibited root elongation of WT but not of *BdPP2CA6*-overexpression plants (**Figures 5A,E**). Fresh weights and root lengths were evaluated and the average fresh weight and root length of *BdPP2CA6*-overexpression plants were higher than that of WT and VC at 150 mM NaCl concentration (**Figures 5G,H**). In addition, growth of 2-week-old seedlings under salinity treatment was measured. Two-week-old WT and *BdPP2CA6*-overexpression plants growing on MS medium were transferred to Hoagland solution supplemented with 0 or 150 mM NaCl. Most WT plants wilted after 1 day, while most BdPP2CA6-overexpression transgenic *Arabidopsis* remained green, indicating that *BdPP2CA6* is involved in responses to salinity stress at the early growing stage (**Figure 7B**).

For NaCl treatment, 3-week-old WT, VC and *BdPP2CA6*-overexpression plants growing in soil were watered with 150 mM NaCl for 30 days. Most WT plants turned purple or wilted whereas most transgenic plants remained green (**Figure 7A**). Survival rates of transgenic seedlings were measured 35 days post NaCl treatment and were significantly higher than that of WT and VC plants (**Figure 7C**). As we found that overexpression of *BdPP2CA6* could promote ABA-mediated stomatal closure, we assumed that *BdPP2CA6*-overexpression plants might be able to withhold more water to cope with external salinity through stomatal closure. To confirm this possibility, we measured stomatal apertures of leaves from WT and *BdPP2CA6*-overexpression plants under NaCl treatment. Under normal condition, the differences between WT and *BdPP2CA6*-overexpression plants were not significant, the stomatal aperture indices of transgenic plants after salinity treatment decreased more rapidly than that of WT, indicating that transgenic plants could withhold more water to cope with salinity stress (**Figure 6**).

### Overexpression of *BdPP2CA6* Increases K^+^/Na^+^ Ratio under Salinity Stress

A higher K^+^/Na^+^ ratio is important for plants to cope with salinity stress (Zhu, 2003). To investigate whether overexpression of *BdPP2CA6* could affect Na^+^ and K^+^ accumulations in plants, the Na^+^ and K^+^ contents were measured. No significant difference in Na^+^ contents was observed between WT and transgenic plants under normal condition or NaCl treatment. Whereas K^+^ contents of transgenic plants were significantly higher than that of WT under both normal condition and NaCl treatment (**Figures 7I–K**). The results demonstrated that overexpression of *BdPP2CA6* increased K^+^ accumulation, thus increasing the ratio of K^+^/Na^+^ under salinity stress condition.

### Overexpression of *BdPP2CA6* Increases Chlorophyll, Proline Content, and Decreased H_2_O_2_, MDA, and IL Level under Salinity Stress

Several physiological indices including IL, H_2_O_2_, proline, chlorophyll, and MDA contents were measured to investigate the physiological mechanisms in *BdPP2CA6*-overexpression plants in response to salinity stress. *BdPP2CA6*-overexpression plants exhibited lower contents of IL, MDA, and H_2_O_2_, and higher levels of chlorophyll and proline compared with WT under NaCl treatment (**Figures 7D–H**). The results suggested that transgenic seedlings suffered less membrane damage and intracellular oxidative stress, therefore were more tolerant to external salinity stress.

### BdPP2CA6 Interacts with Both AtSnRK2 and BdSnRK2

**FIGURE 8:**
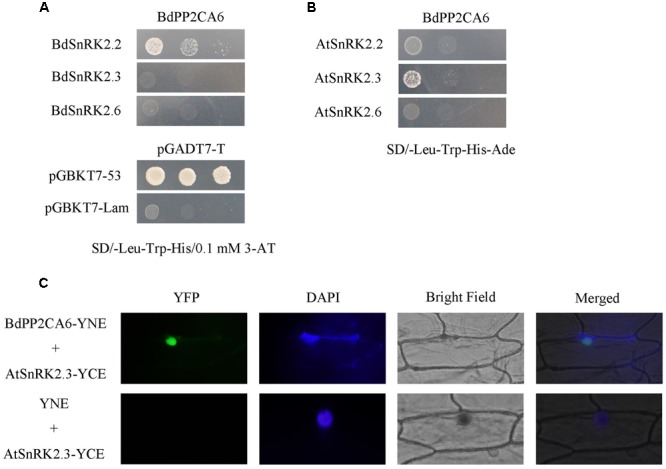
**Interaction analyses of BdPP2CA6-BdSnRKs and BdPP2CA6-AtSnRKs. (A)** pGBKT7-BdPP2CA6 and pGADT7-BdSnRKs were co-transformed and transformed yeast cells were cultivated on selective medium SD/-Trp-Leu-His/0.1 μM 3-AT, respectively. Interactions between SV40-T and p53 or SV40-T and human lamin C were set as positive or negative control, respectively. Yeast strains were assessed by different dilution rates (1, 1/10, and 1/100). **(B)** pGBKT7-BdPP2CA6 and pGADT7-BdSnRKs were co-transformed and transformed yeast cells were cultivated on nutritional selective medium SD/-Trp-Leu-His-Ade. **(C)** BiFC visualization of BdPP2CA6 interaction with AtSnRK2.3 transiently expressed in onion epidermal cells. DAPI staining for the nucleus was performed after YFP and bright field visualization. AtSnRK2.3-YCE and YNE was set as negative control.

To further understand the molecular mechanisms of BdPP2CA6 function in abiotic stress tolerance, interactions between BdPP2CA6 and AtSnRK2/BdSnRK2 were investigated. SnRK2.2/2.3/2.6 were selected as potential targets because SnRK2.2/2.3/2.6 are essential in ABA signaling and interact with several clade A PP2Cs in *Arabidopsis* (Fujita et al., 2009; Nakashima et al., 2009). We chose SD/-Trp-Leu-His/0.1 μM 3-AT to assess growth status of yeast cells co-transformed with BdPP2CA6 and BdSnRKs because they were not able to grow on SD/-Trp-Leu-His-Ade. The results showed that BdPP2CA6 had a mild interaction with BdSnRK2.2 but had a strong interaction with AtSnRK2.3 (**Figures 8A,B**). This strong interaction between BdPP2CA6 and AtSnRK2.3 was further confirmed by BiFC assay (**Figure 8C**).

### Altered Expressions of Related Stress-Responsive Genes in *BdPP2CA6*-Overexpression Plants

**FIGURE 9:**
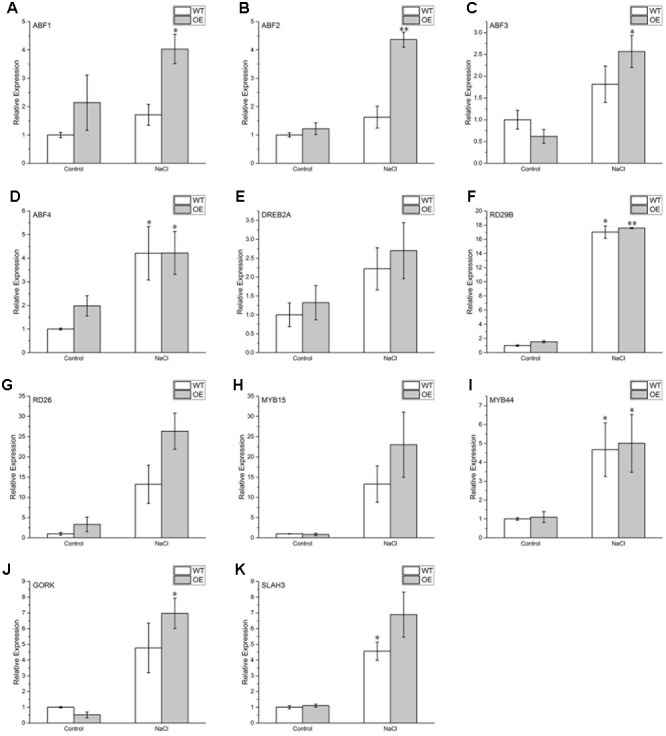
**Expression pattern of relevant genes in WT and *BdPP2CA6*-overexpression seedlings. (A)** *ABF1*; **(B)** *ABF2*; **(C)** *ABF3*; **(D)** *ABF4*; **(E)** *DREB2A*; **(F)** *RD29B*; **(G)** *RD26*; **(H)** *MYB15*; **(I)** *MYB44*; **(J)** *GORK*; **(K)** *SLAH3*. Seedlings of WT and OE were treated with 150 mM NaCl for 10 days and total RNAs were extracted from leaves. Data are means ± SE of three replicates. Significances of differences are indicated as ^∗^*P* < 0.05, ^∗∗^*P* < 0.01.

To identify molecular events involved in BdPP2CA6-mediated signal pathway, expressions of several ABA- and stress-responsive genes were investigated by qRT-PCR technique. The results showed that certain genes (*ABF2, ABF3, DREB2A, MYB15, RD26, GORK, and SLAH3*) were significantly induced by salinity stress and were induced to higher expression levels in transgenic plants than WT while expression levels of *MYB44, RD29B*, and *ABF4* were not significantly different between transgenic plants and WT under salinity stress (**Figure 9**). These results imply that BdPP2CA6 positively regulate the response to salinity stress by modulating expression of diverse stress-related genes in *Arabidopsis* plants.

## Discussion

Abscisic acid is a key phytohormone in abiotic stress response, and understanding regulatory mechanism of ABA signaling is therefore very important. The ABA-PYL-PP2C-SnRK2 signal pathway has been demonstrated in *Arabidopsis* (Fujii et al., 2009). Here, we identified *PYLs* and *PP2CAs* in *B. distachyon* to investigate their functions in ABA signaling as gene function analysis of monocot model plant species *B. distachyon* can help the similar researches being conducted in other important cereal plant species such as wheat. In our study, we found that many interactions between BdPYLs and BdPP2CAs were dependent on the presence of ABA in yeast two-hybrid assay. Strong interaction between BdPYL11 and BdPP2CA6 was confirmed *in vivo* by BiFC assay, therefore we focused on functional analysis of BdPP2CA6 (**Figures 2** and **4A**). Moreover, BdPP2CA6 interacts with BdSnRK2.2 and AtSnRK2.3, suggesting the participation of BdPP2CA6 in core ABA signaling (**Figure 8**).

The subcellular localization of BdPP2CA6 is different from those of clade A PP2Cs in *Arabidopsis*, but consistent with several clade A PP2Cs in rice (Kim et al., 2012; Singh et al., 2015). Our studies showed that BdPP2CA6 and complexes consisting of BdPP2CA6 and its interacting partners, BdPYL11 or AtSnRK2.3 were localized to the nucleus (**Figures 4** and **8C**). This indicates that BdPP2CA6 recruits BdPYL11 to the nucleus and BdPYL11 might function as a nuclear ABA receptor.

We found that overexpression of *BdPP2CA6* in *Arabidopsis* enhances ABA sensitivity (**Figure 5**). Moreover, overexpression of *BdPP2CA6* in *Arabidopsis* promotes stomatal closure under both ABA and NaCl treatments (**Figure 6**). Accumulation of ABA under stress conditions leads to stomatal closure, thus conserving water to survive abiotic stress such as drought (Schroeder et al., 2001; Wasilewska et al., 2008; Kim et al., 2010; Lee and Luan, 2012). In our study, *BdPP2CA6* overexpression plants showed enhanced tolerance to salinity stress. We suppose that ABA-dependent enhanced stomatal closure of *BdPP2CA6* overexpression plants attributes to better tolerance under salinity treatment (**Figure 6**).

A higher K^+^/Na^+^ ratio is important for plants to maintain cellular metabolism and to survive under salinity stress (Zhu, 2003). Higher K^+^/Na^+^ ratio in *BdPP2CA6* overexpressing plants enhances salinity stress tolerance to plants. Regulation of K^+^ channel activity by protein kinases and phosphatases could control K^+^ transport during stresses (Cherel, 2002; Lan et al., 2011). BdPP2CA6 might enhance salinity stress tolerance by regulating K^+^ channels activity.

To gain a further insight into how BdPP2CA6 regulates gene expression under salinity stress, expressions of some stress-related genes were detected. Previous studies have revealed many positive regulators of ABA and stress signaling pathways. ABF1, ABF2, ABF3, and ABF4 are predominant TFs downstream of SnRK2.2/2.3/2.6 in ABA signal pathway, responding to osmotic stresses at vegetative growth stage (Fujita et al., 2005, 2013; Abdeen et al., 2010; Yoshida et al., 2010, 2015). Beside AREB/ABF TFs, many other TFs have been reported to be involved in ABA-mediated gene expression. For instance, DREB2A, MYB15, MYB44, and RD26 are positive regulators of ABA signaling and are strongly induced by osmotic stress (Nakashima et al., 2000; Fujita et al., 2004; Jung et al., 2008; Ding et al., 2009). Higher expression levels of some stress-related genes (*ABF2, ABF3, ABF4, DREB2A, MYB15*, and *RD26*) in *BdPP2CA6* overexpression plants than in WT under salinity treatment are contributive to enhanced salt tolerance.

Previous studies show that several clade A PP2Cs in *Arabidopsis* negatively regulate ABA and stress signaling pathways (Merlot et al., 2001; Saez et al., 2004, 2006; Zhang et al., 2013). In our study, BdPP2CA6 was found to be a positive regulator in both ABA and stress signaling pathways. To uncover how BdPP2CA6 functions is an interesting task. By now, few studies have demonstrated that the clade A PP2Cs could contribute to stress tolerance. Tobacco plants overexpressing *ZmPP2C2* are highly tolerant to cold stress (Hu et al., 2010). Overexpression of *OsPP108* in *Arabidopsis* enhanced high salt and mannitol stress tolerance at stages of seed germination, early development, and adult (Singh et al., 2015). Our finding, that BdPP2CA6 confers salinity tolerance to transgenic *Arabidopsis* plant seedlings, is consistent with the reported results that PP2C genes from monocot plants confer stress tolerance to transgenic plants. However, the mechanism underlying how BdPP2CA6 regulates ABA signaling and stress signaling in *Arabidopsis* is still is unclear, and we cannot rule out that the native function in *Brachypodium* could be different. Previous studies reveal that abi1-1, a mutant of ABI1, lacks PYR/PYL/RCAR-binding ability and constitutively inactivates SnRK2s even in the presence of PYR1 and ABA (Ma et al., 2009; Park et al., 2009; Umezawa et al., 2009). In our study, BdPP2CA6 might share some similarity with the phenotype of abi1-1 but in a converse way. BdPP2CA6 can interact with BdPYL11 in the absence of ABA (**Figure 4B**). Though interaction between BdPP2CA6 and AtSnRK2.3 is confirmed in the BiFC assay, BdPP2CA6 might not inhibit activity of AtSnRK2.3 properly like its homologs do in *Arabidopsis*. Moreover, we tried to confirm the interaction between BdPP2CA6 and BdSnRK2.2 by using BiFC but failed to observe fluorescence signal, suggesting that BdPP2CA6 interacts with BdSnRK2.2 weakly. We were curious if there is any other SnRK2 that could interact with BdPP2CA6 apart from these three commonly known ABA related SnRK2s (SnRK2.2/2.3/2.6), so we cloned the rest SnRK2s from both *Arabidopsis* and *Brachypodium* to carry out yeast-two-hybrid assay and we failed to detect any interaction signal between BdPP2CA6 and SnRK2s (data not shown). Based on above-mentioned observation, we assume that SnRK2s are not the key regulators at downstream of BdPP2CA6. Previous studies reveal that PP2CA specifically interacts with CIPK6 and directly interacts with the CIPK6 kinase domain to inhibit AKT1 activation induced by CIPK6 (Lan et al., 2011). Therefore, the function diversity of clade A PP2Cs and the fact that clade A PP2Cs regulate both ABA-PYL-PP2C-SnRK2 signal pathway and CBL-CIPK-PP2CA network give us new insight into the function mechanism of BdPP2CA6. We also assume that BdPP2CA6 might have undiscovered substrates that function in ABA and stress signaling pathway to confer enhanced stomatal closure, with increased expressions of some stress-responsive genes, and higher K^+^/Na^+^ ratio to the *BdPP2CA6*-overexpressing *Arabidopsis* plant seedlings. On one hand, BdPP2CA6 participates in the classic ABA-PYL-PP2C-SnRK2 core signal pathway but with less inhibition to SnRK2s. On the other hand, BdPP2CA6 positively regulate ABA and stress signaling pathways through certain uncovered substrates. The latter is predominate, therefore overexpression of *BdPP2CA6* in *Arabidopsis* exhibits hypersensitivity to ABA and tolerance to salinity stress.

## Conclusion

BdPP2CA6 is a clade A PP2C from *B. distachyon* and unlike most other clade A PP2Cs in *Arabidopsis*, it positively regulates ABA signaling and salinity stress signaling. *BdPP2CA6* overexpression in *Arabidopsis* leads to hypersensitivity to ABA at early growing stage and enhanced salinity tolerance at both early developing stage and adult stage through ABA-dependent pathway. These findings give us insight into cross talk between ABA and abiotic stress signaling.

## Author Contributions

GH, GY, JC, FZ, and QW designed the experiments. FZ and QW participated in all experiments, analyzed results and wrote the manuscript. JS performed the physiological experiments. XJ constructed the vectors. YH, QL, and YZ performed the qRT-PCR assay. GH, GY, JC, FZ, and YW revised the manuscript. All authors read and approved the manuscript.

## Conflict of Interest Statement

The authors declare that the research was conducted in the absence of any commercial or financial relationships that could be construed as a potential conflict of interest.

## Footnotes

1 https://www.arabidopsis.org

2 http://plants.ensembl.org/Brachypodium_distachyon/Info/Index

3 http://pfam.xfam.org

4 http://bioinformatics.psb.ugent.be/plaza

5 http://bioinformatics.psb.ugent.be/webtools/plantcare/html
