# Supplementary material for: Brachypodium distachyon BdPP2CA6 Interacts with BdPYLs and BdSnRK2 and Positively Regulates Salt Tolerance in Transgenic Arabidopsis
Source: Front Plant Sci. 2017 Feb 28;8:264. doi: 10.3389/fpls.2017.00264 (PMC5329023; doi:10.3389/fpls.2017.00264)
Supplement: Supplementary file 4 [file Image_1.PDF]

A

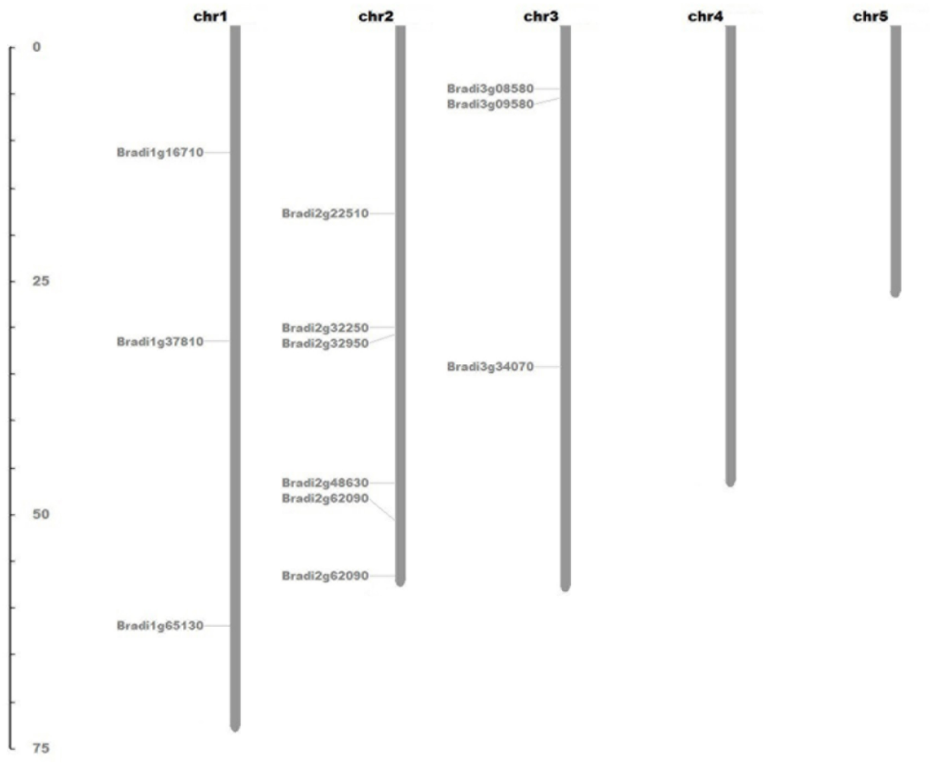

B

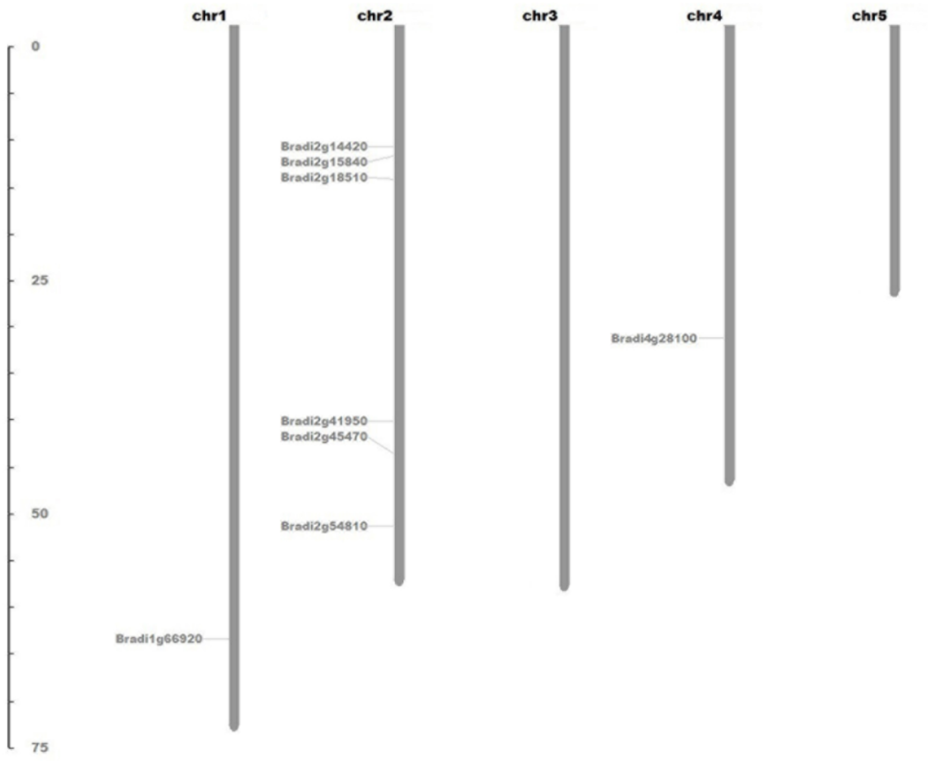

Supplementary Figure S1. Chromosomal locations of *BdPYLs* (A) and *BdPP2CAs* (B). The scale bar represents the megabases (Mb) of the genome.
