# Supplementary material for: Brachypodium distachyon BdPP2CA6 Interacts with BdPYLs and BdSnRK2 and Positively Regulates Salt Tolerance in Transgenic Arabidopsis
Source: Front Plant Sci. 2017 Feb 28;8:264. doi: 10.3389/fpls.2017.00264 (PMC5329023; doi:10.3389/fpls.2017.00264)
Supplement: Supplementary file 5 [file Image_2.PDF]

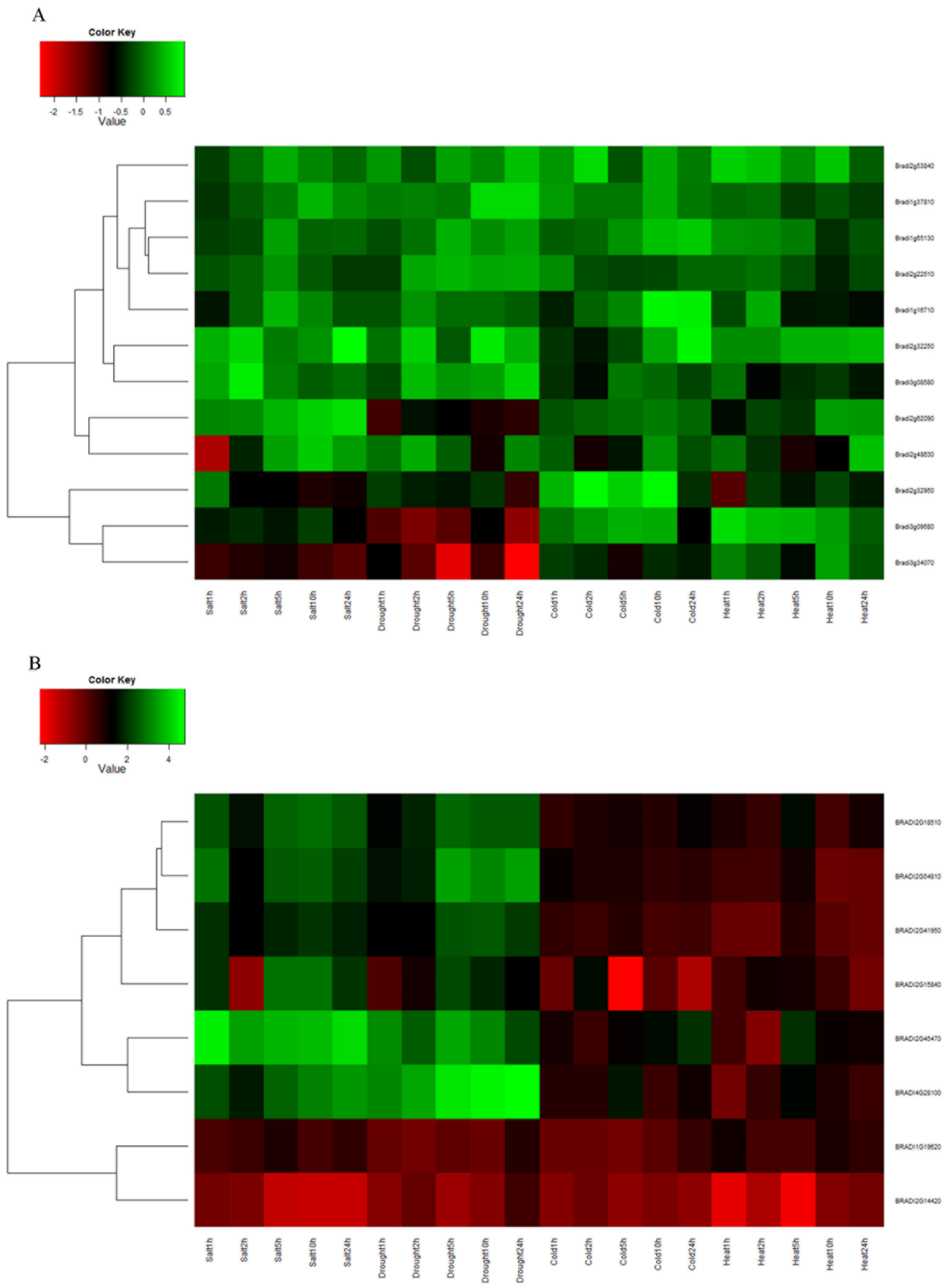

Supplementary Figure S2. Expression profiles (in log_2 based fold change) of *BdPYLs* (A) and *BdPP2CAs* (B) under four abiotic stress conditions.
